# The obesity paradox in critically ill patients: a causal learning approach to a casual finding

**DOI:** 10.1186/s13054-020-03199-5

**Published:** 2020-08-05

**Authors:** Alexander Decruyenaere, Johan Steen, Kirsten Colpaert, Dominique D. Benoit, Johan Decruyenaere, Stijn Vansteelandt

**Affiliations:** 1 Department of Internal Medicine and Pediatrics, Ghent University, C. Heymanslaan 10, 9000 Ghent, Belgium; 2 Department of Medical Oncology, Ghent University Hospital, Ghent, Belgium; 3 Department of Intensive Care Medicine, Ghent University Hospital, Ghent, Belgium; 4 Department of Nephrology, Ghent University Hospital, Ghent, Belgium; 5 Department of Applied Mathematics, Computer Science and Statistics, Ghent University, Ghent, Belgium; 6 Department of Medical Statistics, London School of Hygiene and Tropical Medicine, London, UK

**Keywords:** Obesity, Paradox, Mortality, Causality, Confounding, Selection bias, Machine learning, Super learning, Targeted learning

## Abstract

**Background:**

While obesity confers an increased risk of death in the general population, numerous studies have reported an association between obesity and improved survival among critically ill patients. This contrary finding has been referred to as the obesity paradox. In this retrospective study, two causal inference approaches were used to address whether the survival of non-obese critically ill patients would have been improved if they had been obese.

**Methods:**

The study cohort comprised 6557 adult critically ill patients hospitalized at the Intensive Care Unit of the Ghent University Hospital between 2015 and 2017. Obesity was defined as a body mass index of ≥ 30 kg/m^2^. Two causal inference approaches were used to estimate the average effect of obesity in the non-obese (AON): a traditional approach that used regression adjustment for confounding and that assumed missingness completely at random and a robust approach that used machine learning within the targeted maximum likelihood estimation framework along with multiple imputation of missing values under the assumption of missingness at random. 1754 (26.8%) patients were discarded in the traditional approach because of at least one missing value for obesity status or confounders.

**Results:**

Obesity was present in 18.9% of patients. The in-hospital mortality was 14.6% in non-obese patients and 13.5% in obese patients. The raw marginal risk difference for in-hospital mortality between obese and non-obese patients was − 1.06% (95% confidence interval (CI) − 3.23 to 1.11%, *P* = 0.337). The traditional approach resulted in an AON of − 2.48% (95% CI − 4.80 to − 0.15%, *P* = 0.037), whereas the robust approach yielded an AON of − 0.59% (95% CI − 2.77 to 1.60%, *P* = 0.599).

**Conclusions:**

A causal inference approach that is robust to residual confounding bias due to model misspecification and selection bias due to missing (at random) data mitigates the obesity paradox observed in critically ill patients, whereas a traditional approach results in even more paradoxical findings. The robust approach does not provide evidence that the survival of non-obese critically ill patients would have been improved if they had been obese.

## Background

Obesity is a chronic disease associated with cardiovascular disease, chronic kidney disease, diabetes mellitus, some cancers, and musculoskeletal disorders. It has become one of the most important public health problems in many high- and middle-income countries, entailing a heavy economic burden [1–3]. The prevalence of obesity is steadily increasing worldwide and, on average, approximately one in five patients admitted to the intensive care unit (ICU) is obese [4, 5]. Although obesity confers an increased risk of morbidity and mortality in the general population and poses additional challenges that may compromise prognosis in critically ill patients, a growing body of literature has found an association between obesity and improved ICU outcomes, including lower mortality. This contrary finding has been referred to as the obesity paradox [4–11].

While some authors postulate underlying pathophysiologic mechanisms to support its biological plausibility [4, 9, 10, 12, 13], others remain skeptical and provide methodological explanations. The first potential type of bias is confounding bias. Confounding factors of the obesity-mortality relationship include age, sex, ethnicity, smoking status, alcohol consumption, income, education, physical activity, and dietary pattern, among others [14]. Failure to adequately control for confounding will result in bias [15]. Bias may in particular arise as a consequence of reverse causation, whereby pre-existing disease leads to unintended weight loss and higher mortality, making obesity appear protective [16, 17].

**Fig. 1:**
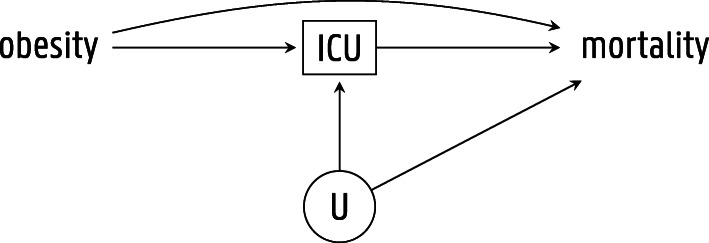
Causal diagram representing causal relations between obesity, ICU admission, and mortality. The circle around variable *U* indicates that it has not been measured. The box around variable ICU indicates that the analysis conditions on it by design, since the study cohort only comprised patients admitted to the intensive care unit. This causal diagram has been simplified for the purpose of illustrating collider stratification bias. ICU, intensive care unit; *U*, unmeasured factors

Secondly, studies restricted to patients with an obesity-related disease may suffer from so-called collider stratification bias. The causal diagram presented in Fig. 1 depicts the causal relations between obesity, ICU admission, and mortality. Obesity may lead to earlier ICU admission, as clinicians tend to consider obese patients at higher risk of worse outcome [4, 12]. The node *U* represents common causes of ICU admission and mortality that are usually unmeasured or difficult to quantify. Examples include perceived reversibility of the acute illness, anticipated quality of life, patient wishes, ceiling of care, peer standards, and bed occupancy status [18, 19]. Because ICU admission may be affected by both obesity as well as these unmeasured prognostic factors of mortality, restriction of the study population to patients admitted to the ICU may induce collider stratification bias, a form of selection bias. In particular, non-obese patients will generally have more potent risk factors *U* for mortality. Indeed, the presence of more potent risk factors (and not the absence of obesity) may explain why these patients got admitted to the ICU [17, 20].

Other potential types of bias include body mass index (BMI) group misallocation by estimating rather than actually measuring height and weight in critically ill patients [21], treatment bias due to closer monitoring and different treatment of obese patients [4, 22, 23], and publication bias due to selective reporting of unusual findings such as the obesity paradox.

An important clinical question that arises from the obesity paradox is whether the survival of non-obese critically ill patients would have been improved if they had been obese. This demands a causal inference approach. While the necessity of adequate confounding control for valid causal inference has been recently emphasized by the editors of many critical care journals, no recommendations have been made on which method to apply [24]. This study purported to address the aforementioned clinical question using two different approaches: a traditional approach that used regression adjustment for confounding and a more robust approach that used state-of-the-art machine learning techniques within a causal inference framework along with missing data imputation.

## Methods

### Study design and data collection

**Fig. 2:**
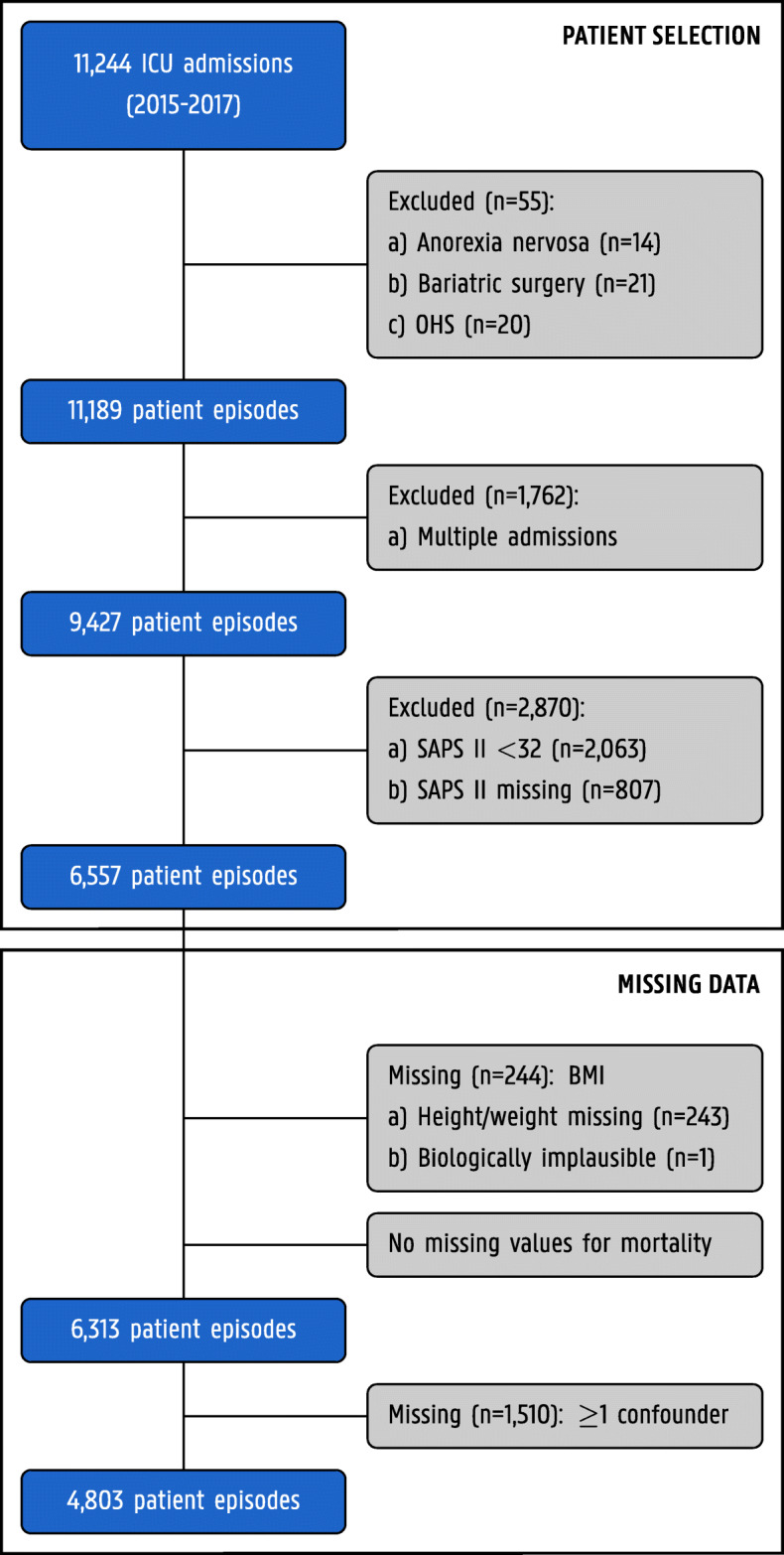
Flow diagram of the study cohort. BMI, body mass index; OHS, obesity hypoventilation syndrome; SAPS II, Simplified Acute Physiology Score II

This retrospective study focused on the relationship between obesity and in-hospital mortality in adult (≥ 16 years) critically ill patients consecutively hospitalized at the ICU of the Ghent University Hospital between January 1, 2015, and December 31, 2017. During the study period 11,244 adult patients were admitted to the ICU. Fifty-five patients were excluded from the analysis because their reason for ICU admission was directly related to underweight or obesity. In case of multiple ICU admissions during the study period (*n* = 1762), only the first was considered. Patients with a Simplified Acute Physiology Score II (SAPS II) of < 32 points (first quartile, *n* = 2063) or missing SAPS II (*n* = 807) at ICU admission were discarded in order to exclude patients who were admitted to the ICU for the sole purpose of monitoring and in whom critical illness was less likely to be present. The final study cohort comprised 6557 patients. A flow diagram is presented in Fig. 2.

Obesity was the exposure of interest and was dichotomously defined as a BMI of ≥ 30 kg/m^2^ according to the classification of the National Institutes of Health and the World Health Organization [1, 25]. A sensitivity analysis was also performed using a BMI cut-off value of ≥ 25 kg/m^2^ and ≥ 35 kg/m^2^. The patient’s height and weight were usually estimated at ICU admission by critical care nurses. A BMI of < 10 or > 80 kg/m^2^ was considered as biologically implausible, thus consequently attributed to input error and handled as missing value. In-hospital mortality was used as outcome variable.

The following potential confounders of the obesity-mortality relationship were extracted or derived from the prospectively maintained Intensive Care Information System database (GE Healthcare Centricity® Critical Care): age, sex, ethnicity, smoking status, alcohol consumption, physical activity, hypothyroidism, chronic glucocorticoid therapy, solid malignancy, hematologic malignancy, dementia, human immunodeficiency virus infection or acquired immunodeficiency syndrome, and calendar time (in years since start of study). As annual income was not available at the patient level, the median annual income (net taxable income in 2015 as provided by Statbel [26]) per statistical sector to which the patient belongs based on home address was used as proxy variable. Variables related to obesity or ICU stay were also obtained. Obesity-related conditions included Charlson comorbidity index, cardiovascular disease, hypertension, diabetes mellitus, chronic kidney disease, chronic liver disease, and chronic pulmonary disease. Variables related to ICU stay included SAPS II within 36 h after ICU admission, type of admission, and ICU mortality. The data on these sociodemographic characteristics and comorbidities contained in the Intensive Care Information System database were inputted manually at ICU admission by critical care physicians. Data discrepancies were resolved by expert opinion or by reviewing the electronic health record.

The estimand of interest was the difference in in-hospital mortality in non-obese patients if all had been obese versus as observed (i.e., if they stayed non-obese). This corresponds with the average treatment effect in the untreated (ATU), which is the marginal causal risk difference in the subpopulation that was not exposed (to obesity at ICU admission). We considered this effect to be of particular interest because it is less susceptible to selection bias. The reason is that non-obese patients would likely also have been admitted to the ICU and have had a SAPS II of ≥ 32 points at ICU admission if they had been obese (so that their selection is not related to their obesity status). By contrast, this seems less plausible to hold for obese patients if they had been non-obese, making the average treatment effect in the treated (and also the overall average treatment effect) more prone to selection bias. Identifying the effect of obesity on in-hospital mortality in non-obese patients requires adequate confounding control [15]. In the following two sections, we describe the two approaches that we considered for this.

### Traditional approach

In line with common approaches [7, 8], a logistic regression model was fitted with in-hospital mortality as the outcome, obesity as a dichotomous covariate, and the main effects of the 14 confounders as other covariates. Model selection procedures were not performed to reduce the risk of residual confounding, leaving all variables in the model. An estimate of the ATU was obtained by direct standardization, also known as G-computation [15, 27]. First, the logistic regression model was used to predict the outcome for each patient as if the patient had been obese and the outcome as if the patient had been non-obese. These predictions were then averaged only across patients who were non-obese. The difference between both averages gave an estimate of the ATU.

This approach has several limitations. First, logistic regression assumes that the log odds of in-hospital mortality are linear in the covariates. Incorrect modeling of the true relationship between confounders and outcome leaves residual confounding bias. This is a major concern, especially when there is little overlap in the confounder values between obese and non-obese patients, for then regression-based estimators can severely rely on extrapolation, without this being visible from the results. For instance, that extrapolations are being made is only subtly hinted at via slightly increased standard errors, thereby possibly leaving an optimistically precise yet biased estimator of the causal effect. Additionally, regression models require complete cases of the data, discarding cases with at least one missing value. Complete case analysis is, however, inefficient and can be biased when missingness is informative [15].

### Robust approach

In view of the foregoing concern about parametric model misspecification, the one-step targeted maximum likelihood estimation (TMLE) framework was used to enable the use of machine learning methods with the aim to reduce bias against model misspecification, while ensuring that valid confidence intervals for the treatment effect can be constructed [27–31]. Here, machine learning was based on a so-called super learning procedure. This is an ensemble method that selects the optimally weighted combination of multiple candidate algorithms by applying a metalearning algorithm that minimizes the cross-validated risk associated with chosen prediction error loss functions. The resulting super learner is designed to perform as well as or better than the best-fitting candidate algorithm (in large sample sizes) [27, 29, 32, 33]. In this study, super learning with stratified 10-fold cross-validation was performed using following library of 15 candidate algorithms: null estimator (unconditional mean); main effects logistic regression model; stepwise logistic regression model; 5 penalized regression models using elastic net with mixing parameter of 0 (ridge penalty), 0.25, 0.50, 0.75, or 1.0 (lasso penalty); random forests; extreme gradient boosting; support vector machines; Bayesian additive regression trees model; and 3 general additive models with polynomial terms to the second, third, or fourth degree. Three super learners for the outcome mechanism and for the exposure mechanism were created by applying the squared error (L2) loss, log loss, and the rank loss functions as metalearning algorithm. Details of the super learning procedure, including the hyperparameter settings of each candidate algorithm, the optimal super learner weights that minimized the corresponding loss function, and the performance measures obtained by nested cross-validation, are presented in Additional file 1. The best performance for the data at hand was shown for the log loss super learners, which were therefore used in the subsequent TMLE procedure. Truncation levels of 2.5% and 97.5% were used for extreme inverse probability weights. Standard errors for the TMLE were calculated based on the influence curve.

TMLE combined with super learning reduces model misspecification bias [27, 29], but it does not tackle the potential problem of selection bias due to missing data. Therefore, multiple imputation by chained equations (MICE) was used to impute missing values. MICE is an iterative algorithm based on fully conditional specification, where the imputation model is specified separately for each incomplete variable in function of all other (possibly incompletely) measured variables. It relies on the missing at random assumption, which states that the probability of a value being missing does not depend on the unobserved data conditional on the observed data [34]. Specification of the imputation model is given in Additional file 2. The number of imputations was set to 50. Each imputed dataset was analyzed separately using the one-step TMLE and the log loss super learner for the outcome and exposure mechanism. The separate estimates and variances for each of the imputed datasets were pooled into an overall estimate and variance using Rubin’s rule [35].

Data analysis was performed in R version 3.3.2 using the *stdReg* version 3.0.0, *SuperLearner* version 2.0-24, *rBayesianOptimization* version 1.1.0, *tmle* version 1.3.0-2, and *mice* version 3.6.0 packages [36].

## Results

### Baseline characteristics

The final study cohort comprised 6557 patients. Obesity was present in 18.9% of patients. The respective ICU and in-hospital mortality was 9.7% and 14.4% in the overall cohort, 9.8% and 14.6% in non-obese patients, and 9.1% and 13.5% in obese patients. The raw marginal risk difference for in-hospital mortality between obese and non-obese patients was − 1.06% (95% confidence interval (CI) of − 3.23 to 1.11%, *P* = 0.337). These findings are consistent with the obesity paradox. Baseline characteristics are summarized in Tables 1 and 2.

**Table 1:** Baseline characteristics (continuous variables)

Variable	Total (***n*** = 6557)		Non-obese (***n*** = 5121)		Obese (***n*** = 1192)	
	Mean (SD)	Median (Q1–Q3)	Mean (SD)	Median (Q1–Q3)	Mean (SD)	Median (Q1–Q3)
**BMI** (kg/m^2^)	26.2 (5.2)	25.6 (23.0–28.8)	24.4 (3.2)	24.6 (22.4–26.9)	34.1 (4.6)	32.9 (31.1–35.4)
**Age** (years)	64.1 (15.3)	67.0 (56.0–75.0)	63.8 (15.8)	67.0 (55.0–75.0)	65.3 (12.1)	67.0 (59.0–74.0)
**Income** (€1000/year)	25.9 (4.1)	26.0 (23.2–28.6)	26.0 (4.0)	26.0 (23.5–28.6)	25.7 (4.2)	25.8 (22.9–28.5)
**CCI** (points)	2.0 (2.3)	2.0 (0.0–3.0)	2.0 (2.3)	2.0 (0.0–3.0)	2.1 (2.2)	2.0 (0.0–3.0)
**SAPS II** (points)	59.5 (16.8)	61.0 (44.0–71.0)	59.2 (16.7)	61.0 (44.0–71.0)	61.0 (16.9)	64.0 (45.0–73.0)
**Calendar time** (years)	1.7 (0.9)	1.7 (0.9–2.4)	1.7 (0.9)	1.7 (0.9–2.4)	1.7 (0.9)	1.7 (0.9–2.4)

*BMI* body mass index, *CCI* Charlson comorbidity index, *Q1* first quartile, *Q3* third quartile, *SAPS II* Simplified Acute Physiology Score II, *SD* standard deviation

**Table 2:** Baseline characteristics (categorical variables)

Variable	Total (***n*** = 6557)	Non-obese (***n*** = 5121)	Obese (***n*** = 1192)
	Count (%)	Count (%)	Count (%)
**Sex**			
Male	4196 (64.0)	3297 (64.4)	742 (62.2)
Female	2361 (36.0)	1824 (35.6)	450 (37.8)
**Ethnicity**			
Asian	25 (0.4)	23 (0.4)	2 (0.2)
Black	48 (0.7)	37 (0.7)	11 (0.9)
Caucasian	6154 (93.9)	4825 (94.2)	1112 (93.3)
Other	79 (1.2)	57 (1.1)	19 (1.6)
Missing	251 (3.8)	179 (3.5)	48 (4.0)
**Smoking status**			
Never-smoker	3254 (49.6)	2563 (50.0)	569 (47.7)
Ex-smoker > 1 y	1721 (26.2)	1303 (25.4)	374 (31.4)
Ex-smoker < 1 y	220 (3.4)	172 (3.4)	41 (3.4)
Current smoker	1158 (17.7)	939 (18.3)	174 (14.6)
Missing	204 (3.1)	144 (2.8)	34 (2.9)
**Alcohol consumption**			
Abstinent	3823 (58.3)	3011 (58.8)	686 (57.6)
Social	1832 (27.9)	1409 (27.5)	364 (30.5)
Problematic	714 (10.9)	568 (11.1)	113 (9.5)
Missing	188 (2.9)	133 (2.6)	29 (2.4)
**Physical activity**			
No restriction	3791 (57.8)	3042 (59.4)	626 (52.5)
Limited	2200 (33.6)	1679 (32.8)	439 (36.8)
Chair-ridden	302 (4.6)	207 (4.0)	83 (7.0)
Bed-ridden	167 (2.5)	128 (2.5)	32 (2.7)
Missing	97 (1.5)	65 (1.3)	12 (1.0)
**Hypothyroidism**			
Yes	280 (4.3)	213 (4.2)	60 (5.0)
No	5804 (88.5)	4554 (88.9)	1041 (87.3)
Missing	473 (7.2)	354 (7.0)	91 (7.6)
**Chronic glucocorticoid therapy**			
Yes	444 (6.8)	366 (7.1)	65 (5.5)
No	6031 (92.0)	4702 (91.8)	1117 (93.7)
Missing	82 (1.3)	53 (1.0)	10 (0.8)
**Solid malignancy**			
None	5053 (77.1)	3905 (76.3)	962 (80.7)
Non-metastatic	900 (13.7)	714 (13.9.)	159 (13.3)
Metastatic	514 (7.8)	442 (8.6)	60 (5.0)
Missing	90 (1.4)	60 (1.2)	11 (0.9)
**Hematologic malignancy**			
None	6085 (92.8)	4744 (92.6)	1126 (94.5)
ALL	28 (0.4)	25 (0.5)	3 (0.3)
AML	64 (1.0)	54 (1.1)	9 (0.8)
CLL	34 (0.5)	27 (0.5)	4 (0.3)
MM	35 (0.5)	28 (0.5)	5 (0.4)
NHL	70 (1.1)	59 (1.2)	11 (0.9)
Other	157 (2.4)	128 (2.5)	25 (2.1)
Missing	84 (1.3)	56 (1.1)	9 (0.8)
**Dementia**			
Yes	139 (2.1)	116 (2.3)	18 (1.5)
No	6355 (96.9)	4967 (97.0)	1168 (98.0)
Missing	63 (1.0)	38 (0.7)	6 (0.5)
**HIV/AIDS**			
Yes	43 (0.7)	43 (0.8)	0 (0.0)
No	6427 (98.0)	5020 (98.0)	1182 (99.2)
Missing	87 (1.3)	58 (1.1)	10 (0.8)
**Cardiovascular disease**			
Yes	3235 (49.3)	2442 (47.7)	679 (57.0)
No	3253 (49.6)	2635 (51.5)	507 (42.5)
Missing	69 (1.1)	44 (0.9)	6 (0.5)
**Hypertension**			
Yes	2866 (43.7)	2081 (40.6)	696 (58.4)
No	3603 (54.9)	2981 (58.2)	487 (40.9)
Missing	88 (1.3)	59 (1.2)	9 (0.8)
**Diabetes mellitus**			
Yes	1281 (19.5)	816 (15.9)	421 (35.3)
No	5194 (79.2)	4251 (83.0)	762 (63.9)
Missing	82 (1.3)	54 (1.1)	9 (0.8)
**Chronic kidney disease**			
Yes	1317 (20.1)	974 (19.0)	305 (25.6)
No	5149 (78.5)	4085 (79.8)	877 (73.6)
Missing	91 (1.4)	62 (1.2)	10 (0.8)
**Chronic liver disease**			
Yes	408 (6.2)	312 (6.1)	80 (6.7)
No	6058 (92.4)	4748 (92.7)	1001 (92.4)
Missing	91 (1.4)	31 (1.2)	11 (0.9)
**Chronic pulmonary disease**			
Yes	994 (15.2)	741 (14.5)	225 (18.9)
No	5471 (83.4)	4318 (84.3)	956 (80.2)
Missing	92 (1.4)	62 (1.2)	11 (0.9)
**Type of admission**			
Elective surgery	2727 (41.6)	2094 (40.9)	550 (46.1)
Urgent surgery	989 (15.1)	776 (15.2)	168 (14.1)
Trauma/burns	214 (3.3)	170 (3.3)	22 (1.8)
Monitoring	1503 (22.9)	1198 (23.4)	247 (20.7)
Organ failure	1097 (16.7)	864 (16.9)	202 (16.9)
Missing	27 (0.4)	19 (0.4)	3 (0.3)

*AIDS* acquired immunodeficiency syndrome, *AML* acute myeloid leukemia, *ALL* acute lymphoblastic leukemia, *CLL* chronic lymphocytic leukemia, *HIV* human immunodeficiency virus, *MM* multiple myeloma, *NHL* non-Hodgkin lymphoma, *y* year

### Traditional approach

The results of the multivariate logistic regression model are presented in Table 3. This model indicated that the odds for in-hospital mortality were 20% lower in obese patients compared with non-obese patients with the same level of the confounders that were controlled for (adjusted odds ratio of 0.80, 95% CI 0.64 to 0.99, *P* = 0.048). Using standardization, this translated into a marginal causal risk difference—in particular an ATU—of − 2.48% (95% CI − 4.80 to − 0.15%, *P* = 0.037). This suggested that the in-hospital mortality would be significantly smaller if all non-obese patients had been obese. This regression-based approach thus resulted in even more paradoxical findings, despite presumed adjustment for confounding.

**Table 3:** Multivariate logistic regression model for in-hospital mortality (*n* = 4803)

Variable	***β*** (SE)	OR (95% CI)	***P***
**Intercept**	− 2.32 (0.102)	–	–
**Obesity**	− 0.23 (0.115)	0.80 (0.64–0.99)	0.048
**Age** (standardized)	0.28 (0.054)	1.32 (1.19–1.47)	< 0.001
**Sex, male**	0.10 (0.098)	1.11 (0.91–1.34)	0.30
**Ethnicity**			
Caucasian	reference		
Asian	− 0.04 (0.784)	0.96 (0.21–4.47)	0.96
Black	0.39 (0.575)	1.48 (0.48–4.56)	0.50
Other	− 0.31 (0.448)	0.73 (0.31–1.76)	0.49
**Income** (standardized)	− 0.06 (0.044)	0.95 (0.87–1.03)	0.20
**Smoking status**			
Never-smoker	reference		
Ex-smoker > 1 year	0.02 (0.109)	1.02 (0.82–1.26)	0.87
Ex-smoker < 1 year	0.46 (0.218)	1.59 (1.04–2.44)	0.033
Current smoker	0.18 (0.131)	1.20 (0.93–1.56)	0.16
**Alcohol consumption**			
Abstinent	reference		
Social	− 0.31 (0.109)	0.73 (0.59–0.91)	0.004
Problematic	0.39 (0.139)	1.47 (1.12–1.94)	0.005
**Physical activity**			
No restriction	reference		
Limited	0.55 (0.095)	1.74 (1.45–2.10)	< 0.001
Chair-ridden	1.05 (0.167)	2.87 (2.07–3.98)	< 0.001
Bed-ridden	1.21 (0.214)	3.34 (2.20–5.09)	< 0.001
**Hypothyroidism**	0.03 (0.193)	1.03 (0.70–1.50)	0.89
**Chronic glucocorticoids**	0.28 (0.149)	1.33 (0.99–1.78)	0.056
**Solid malignancy**			
None	reference		
Non-metastatic	− 0.10 (0.125)	0.90 (0.71–1.15)	0.41
Metastatic	0.23 (0.161)	1.26 (0.92–1.73)	0.15
**Hematologic malignancy**			
None	reference		
Acute lymphoblastic leukemia	0.29 (0.647)	1.33 (0.37–4.73)	0.66
Acute myeloid leukemia	0.88 (0.374)	2.41 (1.16–5.01)	0.019
Chronic lymphocytic leukemia	1.28 (0.440)	2.59 (1.51–8.50)	0.004
Multiple myeloma	0.66 (0.458)	1.93 (0.79–4.75)	0.15
Non-Hodgkin lymphoma	0.41 (0.359)	1.50 (0.74–3.04)	0.26
Other	0.74 (0.223)	2.11 (1.36–3.26)	< 0.001
**Dementia**	0.04 (0.245)	1.04 (0.64–1.68)	0.88
**HIV/AIDS**	− 0.63 (0.619)	0.53 (0.16–1.80)	0.31
**Calendar time** (standardized)	0.05 (0.043)	1.05 (0.96–1.14)	0.26

*AIDS* acquired immunodeficiency syndrome, *CI* confidence interval, *HIV* human immunodeficiency virus, *OR* odds ratio, *SE* standard error

Although only 2.7% of values for exposure and confounders were missing, 1754 (26.8%) patients had at least one missing value and were thereby discarded in the traditional approach. However, the complete cases formed a selective, non-random sample in which the association between obesity and in-hospital mortality became more pronounced. Indeed, the raw marginal risk difference for in-hospital mortality between obese and non-obese patients went from − 1.06% (95% CI − 3.23 to 1.11%, *P* = 0.337) in patients with complete cases for exposure to − 2.25% (95% CI − 4.65 to 0.15%, *P* = 0.067) in patients with complete cases for exposure and confounders. Missingness was equally common in obese and non-obese patients (24.2% versus 23.9%, *P* = 0.828).

Additionally, and somewhat counterintuitively, the above regression adjustment seemed to indicate that the bias arising from measured confounders was towards the null. Indeed, the marginal causal risk difference of − 2.48% was further away from zero than the raw marginal risk difference of − 2.25% in patients with complete cases for exposure and confounders. This suggested that a seemingly protective effect of obesity on in-hospital mortality had been partially masked by worse baseline confounders in obese patients. However, this may also be the result of model misspecification and, hence, of inadequate removal of confounding bias.

### Robust approach

We therefore provided a re-analysis based on TMLE and missing data imputation which, interestingly, mitigated the obesity paradox by yielding an ATU of − 0.59% (95% CI − 2.77 to 1.60%, *P* = 0.599). A summary table of the traditional versus robust approach is given in Table 4. This table also provides the separate impact of performing TMLE or missing data imputation on the marginal causal risk difference. Traditional regression adjustment on the imputed data resulted in a pooled ATU of − 1.46% (95% CI − 3.58 to 0.66%, *P* = 0.178), while performing TMLE on the complete cases (thus without missing data imputation) gave a similar ATU of − 1.52% (95% CI − 3.85 to 0.81%, *P* = 0.201). Only the combination of both techniques attenuated evidence for an obesity paradox. Truncation of the exposure weights was necessary in 0.1% and 0.6% of patients in the TMLE analysis without and with missing data imputation, respectively.

**Table 4:** Summary table of the traditional versus robust causal inference approach

Estimand	Method	Complete cases in A		Complete cases in (A, C)		Imputation for (A, C)	
		Estimate (95% CI)	***P***	Estimate (95% CI)	***P***	Estimate (95% CI)	***P***
**Raw marginal risk difference (unadjusted)**	Regression	− 1.06% (− 3.23 to 1.11)	0.337	− 2.25% (− 4.65 to 0.15)	0.067	− 1.03% (− 3.22 to 1.14)	0.354
**Marginal causal risk difference (ATU)**	Regression + G-computation	**–**	**–**	− 2.48% (− 4.80 to − 0.15)	0.037	− 1.46% (− 3.58 to 0.66)	0.178
	TMLE + super learner	**–**	**–**	− 1.52% (− 3.85 to 0.81)	0.201	− 0.59% (− 2.77 to 1.60)	0.599

With A = obesity and C = age, sex, ethnicity, income, smoking status, alcohol consumption, physical activity, hypothyroidism, chronic glucocorticoid therapy, solid malignancy, hematological malignancy, dementia, human immunodeficiency virus/acquired immunodeficiency syndrome, calendar time

*ATU* average treatment effect in the untreated, *CI* confidence interval, *TMLE* targeted maximum likelihood estimation

A sensitivity analysis was performed by repeating the above analysis for different BMI cut-off values. Details are given in Additional file 3. The raw marginal risk difference for in-hospital mortality between patients with a BMI of ≥ 25 kg/m^2^ (54.8% of patients) and patients with a BMI of < 25 kg/m^2^ was − 1.40% (95% CI − 3.14 to 0.35%, *P* = 0.117). The traditional approach resulted in an ATU of − 2.11% (95% CI − 4.10 to − 0.12%, *P* = 0.038), whereas the robust approach yielded an ATU of − 0.01% (95% CI − 1.81 to 1.79%, *P* = 0.992), thereby debunking this overweight/obesity paradox. Since only a small proportion of patients had a BMI ≥ 35 kg/m^2^ (5.2% of patients), the confidence intervals of the effect estimates were too wide to make a meaningful comparison between the traditional and robust approach in patients with a BMI ≥ 35 kg/m^2^ versus patients with a BMI < 35 kg/m^2^.

## Discussion

The obesity paradox refers to the counterintuitive association between obesity and improved survival rates in critically ill patients and may arise from several methodological pitfalls, including confounding bias and collider stratification bias. The underlying question that clinicians may ask is whether the survival of non-obese critically ill patients would have been improved if they had been obese. This has, to our knowledge, not yet been addressed by prior clinical research and demands a causal inference approach.

In this study, the raw marginal risk difference for in-hospital mortality between obese and non-obese patients admitted to the ICU who have a SAPS II of ≥ 32 points at ICU admission was − 1.06% (95% CI − 3.23 to 1.11%, *P* = 0.337). Two causal inference approaches were used to estimate the ATU: an approach that used traditional regression adjustment for confounding and that assumed missingness completely at random, and a robust approach that used super learning within the TMLE framework along with multiple imputation of missing values under the assumption of missingness at random. The traditional approach resulted in an ATU of − 2.48% (95% CI − 4.80 to − 0.15%, *P* = 0.037), which was an even more paradoxical finding. This may, however, be subject, among others, to selection bias due to missing data and residual confounding bias due to model misspecification. By contrast, the robust approach that combined targeted learning with multiple imputation to deal with both types of biases yielded an ATU of − 0.59% (95% CI − 2.77 to 1.60%, *P* = 0.599) and thereby mitigated the obesity paradox. Thus, this study did not provide evidence that the survival of non-obese critically ill patients would have been improved if they had been obese, nor did it prove that their survival would have been worse.

Nevertheless, caution is warranted in the interpretation of the study findings. First, despite the inclusion of a large number of confounders, residual confounding due to inaccurate proxy variables or unmeasured variables cannot be ruled out. In particular, data on the patient’s dietary pattern and educational level were lacking and may have confounded the obesity-mortality relationship. Additionally, the inclusion of some confounders can be disputed. Indeed, the obesity status measured upon admission to the ICU is the result of a potentially long time-varying process, which makes, for example, control for malignancy potentially not entirely appropriate for eliminating confounding bias, as it may eliminate indirect effects of obesity via malignancy, while also introducing collider stratification bias [16]. Furthermore, it should be noted that 1754 (26.8%) patients were discarded in the traditional approach because of at least one missing value for exposure or confounders.

Secondly, while our analysis focused on the subgroup of non-obese patients to dampen the possible effect of collider stratification bias due to the selection of patients with a SAPS II of at least 32 points who have been admitted to the ICU, such bias in the ATU cannot be entirely ruled out. It may be biased when obesity affects the probability of either ICU admission or SAPS II being ≥ 32 points, as is likely the case. Overcoming the above two problems would necessitate a population cohort study, where individuals’ body weight and the evolution of confounders such as malignancy can be monitored over time [37]. Unfortunately, such data are currently lacking.

A third limitation of the current analysis is that the obtained estimate and its standard error may be subject to residual bias when the dependence of the exposure or outcome on the confounders is too complex to approximate well by super learning at the considered sample sizes [29]. Fourthly, while the MICE algorithm relaxes the implicit missing completely at random assumption of a complete case analysis (as in the first approach), it still relies on the untestable missing at random assumption and on correct specification of the parametric imputation model, which may further bias the TMLE estimator [34]. Finally, although a sensitivity analysis using different BMI cut-off values did not change the interpretation of the results, a possibly non-linear dose-response relationship between BMI and in-hospital mortality among critically ill patients may be inadequately captured by the binary nature of the definition used for obesity.

## Conclusions

A causal inference approach that is robust to residual confounding bias due to model misspecification and selection bias due to missing (at random) data mitigates the obesity paradox observed in critically ill patients, whereas a traditional approach results in even more paradoxical findings. The robust approach does not provide evidence that the survival of non-obese critically ill patients would have been improved if they had been obese.

## Supplementary information

**Additional file 1.** Details of the super learning procedure.

**Additional file 2.** Specification of the imputation model.

**Additional file 3.** Sensitivity analysis using different BMI cut-off values.

## Data Availability

The dataset used and analyzed during the current study are available from the corresponding author on reasonable request.

## Abbreviations

ATU: Average treatment effect in the untreated
BMI: Body mass index
CI: Confidence interval
ICU: Intensive care unit
MICE: Multiple imputation by chained equations
SAPS II: Simplified Acute Physiology Score II
TMLE: Targeted maximum likelihood estimation
