# Supplementary material for: The obesity paradox in critically ill patients: a causal learning approach to a casual finding
Source: Crit Care. 2020 Aug 5;24:485. doi: 10.1186/s13054-020-03199-5 (PMC7405433; doi:10.1186/s13054-020-03199-5)
Supplement: Supplementary file 1 — Additional file 1. Details of the super learning procedure. [file 13054_2020_3199_MOESM1_ESM.docx]

**Additional File 1**

**Hyperparameter settings of the candidate algorithms**

Super learning with stratified 10-fold cross-validation was performed to estimate the conditional mean outcome and exposure using following library of 15 candidate algorithms with the default hyperparameter settings within the *SuperLearner* R package [1] unless otherwise specified: null estimator, main effects logistic regression model, stepwise logistic regression model, 5 penalized regression models using elastic net with mixing parameter of 0 (ridge penalty), 0.25, 0.50, 0.75 or 1.0 (lasso penalty), random forest of 1,000 trees using Bayesian optimization for hyperparameter tuning, extreme gradient boosting using Bayesian optimization for hyperparameter tuning, support vector machine with radial basis kernel, Bayesian additive regression trees model of 100 trees, and 3 general additive models with polynomial terms to the second, third or fourth degree. The null estimator was simply the unconditional mean and had been included as a benchmark algorithm.

Bayesian optimization with Gaussian processes [2] was performed using the *rBayesianOptimization* R package [3] with respect to the cross-validated log loss for the number of variables to possibly split at in each node (lower bound of 3 and upper bound equal to the number of unconverted predictors) and the minimal node size (lower bound of 2% and upper bound of 10% of sample size) in the random forest using 10 seed points and 5 iterations, and for the maximum depth of a tree (lower bound of 3 and upper bound of 10), the minimum sum of instance weight needed in a child (lower bound of 1 and upper bound of 40), the subsample ratio of the training instances (lower bound of 50% and upper bound of 100%), the subsample ratio of predictors when constructing each tree (lower bound of 50% and upper bound of 100%) and the number of iterations (lower bound of 1 and upper bound of 1000 with early stopping after 30 iterations) in extreme gradient boosting using 20 seed points and 10 iterations. Bayesian optimization of the hyperparameters was not performed either for the support vector machine because an initial random grid search for the cost parameter and the inverse kernel width did not lead to substantial changes in the cross-validated log loss, or for the Bayesian additive regression trees model because of the high computational burden.

**Super learner weights**

Three super learners for the outcome mechanism and for the exposure mechanism were created by applying the squared error (L2) loss, log loss and the rank loss functions as metalearning algorithm (henceforth named the L2 loss, log loss and rank loss super learner, respectively). The latter was chosen because in-hospital mortality and obesity were imbalanced events, with rates of approximately 10% to 20%. The Broyden–Fletcher–Goldfarb–Shanno (BFGS) quasi-Newton method with lower bound of 0 and upper bound of 1 was used for box-constrained optimization of the super learner weights [4]. The optimal weights that minimized the corresponding loss function are given in Table A1.

Tree-based methods had the largest weights in all super learner estimates. The random forest had a weight of 50.3%, 54.3% and 40.6% in the L2 loss, log loss and rank loss super learner for the outcome mechanism, respectively, and a weight of 19.8% in the rank loss super learner for the exposure mechanism, whereas it had no weight in the L2 loss and log loss super learner for the exposure mechanism. Extreme gradient boosting received a weight of 11.9%, 13.4% and 21.8% in the L2 loss, log loss and rank loss super learner for the outcome mechanism, respectively, and a weight of 6.2%, 16.0% and 19.9% in the L2 loss, log loss and rank loss super learner for the exposure mechanism, respectively. The Bayesian additive regression trees model had been given a weight of 35.7%, 32.2% and 23.7% in the L2 loss, log loss and rank loss super learner for the outcome mechanism, respectively, and a weight of 87.8%, 62.8% and 38.8% in the L2 loss, log loss and rank loss super learner for the exposure mechanism, respectively. Finally, a weight of 15.4% and 12.1% was given to the general additive model with polynomial terms to the fourth degree in the log loss and rank loss super learner for the exposure mechanism, respectively. The remaining candidate algorithms received little to no weight.

| **Table A1.** Super learner weights. | | | | | | |
| --- | --- | --- | --- | --- | --- | --- |
| **Base**  **algorithm** | **Outcome model** | | | **Exposure model** | | |
|  | **L2 loss SL** | **Log loss SL** | **Rank loss SL** | **L2 loss SL** | **Log loss SL** | **Rank loss SL** |
| **Mean** | 2.15% | 0.08% | 6.61% | 6.04% | 5.77% | 5.28% |
| **LR** | 0.00% | 0.00% | 0.31% | 0.00% | 0.00% | 0.07% |
| **Stepwise LR** | 0.00% | 0.00% | 0.18% | 0.00% | 0.00% | 0.30% |
| **PR (α=1)** | 0.00% | 0.00% | 0.36% | 0.00% | 0.00% | 0.00% |
| **PR (α=0.75)** | 0.00% | 0.00% | 0.19% | 0.00% | 0.00% | 0.10% |
| **PR (α=0.50)** | 0.00% | 0.00% | 0.32% | 0.00% | 0.00% | 0.00% |
| **PR (α=0.25)** | 0.00% | 0.00% | 0.33% | 0.00% | 0.00% | 0.00% |
| **PR (α=0)** | 0.00% | 0.00% | 0.00% | 0.00% | 0.00% | 0.00% |
| **RF** | 50.31% | 54.29% | 40.60% | 0.00% | 0.00% | 19.76% |
| **XGB** | 11.87% | 13.44% | 21.77% | 6.16% | 15.98% | 19.90% |
| **BART** | 35.67% | 32.18% | 23.69% | 87.80% | 62.82% | 38.81% |
| **SVM** | 0.00% | 0.00% | 5.42% | 0.00% | 0.00% | 0.00% |
| **GAM (2°)** | 0.00% | 0.00% | 0.19% | 0.00% | 0.00% | 0.19% |
| **GAM (3°)** | 0.00% | 0.00% | 0.03% | 0.00% | 0.00% | 3.51% |
| **GAM (4°)** | 0.00% | 0.00% | 0.00% | 0.00% | 0.00% | 12.08% |
| Abbreviations: BART, Bayesian additive regression trees; GAM, general additive model; LR, logistic regression; PR, penalized regression; RF, random forest; SL, super learner; SVM, support vector machine; XGB, extreme gradient boosting. | | | | | | |

**Performance of the super learners**

As the super learners are the optimal combination of the cross-validated estimates of the candidate algorithms with respect to the corresponding loss functions, nested cross-validation is needed to assess the performance [5]. An outer stratified 10-fold cross-validation was therefore used to evaluate the super learners created by the inner cross-validation. The performances measures, including the mean squared error, log loss and area under the receiver operating characteristic curve, of all super learners and candidate algorithms are presented in Table A2. Standard errors were calculated based on the influence curve.

The performance measures of the super learners were equivalent to, or at most slightly better than, those of the best candidate algorithms, i.e., the random forest for the outcome mechanism and the Bayesian additive regression trees model for the exposure mechanism. Interestingly, the log loss super learner performed better than the L2 loss and rank loss super learner not only with respect to the cross-validated log loss, but also with respect to both the cross-validated mean squared error and area under the receiver operating characteristic curve. However, there was no significant difference in performance among the different super learners and between the super learners and the best candidate algorithms.

| **Table A2.** 10-fold cross-validated performance measures. | | | | | | |
| --- | --- | --- | --- | --- | --- | --- |
| **Algorithm** | **Outcome model** | | | **Propensity score model** | | |
|  | **MSE (SE)** | **Log loss (SE)** | **AUROC (SE)** | **MSE (SE)** | **Log loss (SE)** | **AUROC (SE)** |
| **L2 loss SL** | 0.1156 (0.0034) | 0.3854 (0.0089) | 0.6613 (0.0112) | 0.1494 (0.0034) | 0.4722 (0.0083) | 0.6066 (0.0102) |
| **Log loss SL** | 0.1155 (0.0034) | 0.3852 (0.0090) | 0.6618 (0.0111) | 0.1493 (0.0034) | 0.4718 (0.0083) | 0.6070 (0.0102) |
| **Rank loss SL** | 0.1157 (0.0034) | 0.3858 (0.0089) | 0.6605 (0.0111) | 0.1494 (0.0034) | 0.4722 (0.0082) | 0.6061 (0.0102) |
| **Mean** | 0.1203 (0.0036) | 0.4048 (0.0091) | 0.5000 (0.0208) | 0.1528 (0.0035) | 0.4836 (0.0082) | 0.5000 (0.0185) |
| **LR** | 0.1171 (0.0035) | 0.3912 (0.0092) | 0.6436 (0.0114) | 0.1519 (0.0035) | 0.4831 (0.0090) | 0.5629 (0.0106) |
| **Stepwise LR** | 0.1173 (0.0035) | 0.3923 (0.0092) | 0.6376 (0.0115) | 0.1518 (0.0035) | 0.4824 (0.0089) | 0.5622 (0.0106) |
| **PR (α=1)** | 0.1168 (0.0035) | 0.3905 (0.0090) | 0.6430 (0.0114) | 0.1517 (0.0035) | 0.4799 (0.0083) | 0.5629 (0.0106) |
| **PR (α=0.75)** | 0.1169 (0.0035) | 0.3906 (0.0090) | 0.6435 (0.0114) | 0.1517 (0.0035) | 0.4798 (0.0083) | 0.5627 (0.0106) |
| **PR (α=0.50)** | 0.1168 (0.0035) | 0.3904 (0.0090) | 0.6439 (0.0114) | 0.1517 (0.0035) | 0.4799 (0.0083) | 0.5624 (0.0106) |
| **PR (α=0.25)** | 0.1168 (0.0035) | 0.3905 (0.0090) | 0.6434 (0.0114) | 0.1517 (0.0035) | 0.4799 (0.0083) | 0.5634 (0.0106) |
| **PR (α=0)** | 0.1169 (0.0035) | 0.3905 (0.0090) | 0.6440 (0.0113) | 0.1517 (0.0035) | 0.4800 (0.0083) | 0.5645 (0.0106) |
| **RF** | 0.1158 (0.0034) | 0.3859 (0.0090) | 0.6597 (0.0112) | 0.1503 (0.0034) | 0.4747 (0.0083) | 0.5982 (0.0101) |
| **XGB** | 0.1172 (0.0035) | 0.3912 (0.0092) | 0.6399 (0.0113) | 0.1504 (0.0034) | 0.4747 (0.0082) | 0.5964 (0.0101) |
| **BART** | 0.1159 (0.0034) | 0.3867 (0.0091) | 0.6577 (0.0113) | 0.1493 (0.0034) | 0.4722 (0.0083) | 0.6077 (0.0102) |
| **SVM** | 0.1205 (0.0036) | 0.4052 (0.0091) | 0.5212 (0.0129) | 0.1528 (0.0035) | 0.4838 (0.0083) | 0.5155 (0.0106) |
| **GAM (2°)** | 0.1166 (0.0034) | 0.3894 (0.0091) | 0.6475 (0.0113) | 0.6475 (0.0113) | 0.4761 (0.0088) | 0.5981 (0.0104) |
| **GAM (3°)** | 0.1165 (0.0034) | 0.3891 (0.0092) | 0.6482 (0.0114) | 0.6482 (0.0114) | 0.4751 (0.0088) | 0.6048 (0.0102) |
| **GAM (4°)** | 0.1165 (0.0034) | 0.3894 (0.0092) | 0.6472 (0.0114) | 0.6472 (0.0114) | 0.4750 (0.0089) | 0.6064 (0.0102) |
| Standard errors for the cross-validated performance measures were calculated based on the influence curve.  Abbreviations: AUROC, area under the receiver operating characteristic curve; BART, Bayesian additive regression trees; GAM, general additive model; LR, logistic regression; MSE, mean squared error; PR, penalized regression; RF, random forest; SE, standard error; SL, super learner; SVM, support vector machine; XGB, extreme gradient boosting. | | | | | | |

**References**

1. Polley E, LeDell E, Kennedy C, Lendle S, van der Laan M. SuperLearner: Super Learner Prediction. R package version 2.0-24. Available at: <https://cran.r-project.org/package=SuperLearner>.
2. Snoek J, Larochelle H, Adams RP. Practical Bayesian optimization of machine learning algorithms. Proceedings of the 25th International Conference on Neural Information Processing Systems. 2012;2;2951-59.
3. Yan Y. rBayesianOptimization: Bayesian Optimization of Hyperparameters. R package version 1.1.0. Available at: [https://cran.r-project.org/package=rBayesianOptimization](https://cran.r-project.org/package=SuperLearner).
4. Byrd RH, Lu P, Nocedal J, Zhu C. A Limited Memory Algorithm for Bound Constrained Optimization. SIAM Journal on Scientific Computing. 1995;16(5):1190-1208.
5. van der Laan M, Rose S. Targeted Learning in Data Science: Causal Inference for Complex Longitudinal Studies. Cham: Springer International Publishing; 2018.
