# Supplementary material for: The obesity paradox in critically ill patients: a causal learning approach to a casual finding
Source: Crit Care. 2020 Aug 5;24:485. doi: 10.1186/s13054-020-03199-5 (PMC7405433; doi:10.1186/s13054-020-03199-5)
Supplement: Supplementary file 2 — Additional file 2. Specification of the imputation model. [file 13054_2020_3199_MOESM2_ESM.docx]

**Additional File 2**

**Multiple imputation by chained equations**

Many modeling techniques require complete cases of the data and thereby discard cases with at least one missing value. Complete case analysis is, however, inefficient and can be biased when missingness is informative. Fortunately, there are several approaches to deal with incomplete multivariate data. Multiple imputation by chained equations (MICE) is an iterative algorithm based on fully conditional specification, where the imputation model is specified separately for each incomplete variable in function of all other (possibly incompletely) measured variables [1].

**Missingness at random**

Specification of the imputation model relies on the missing at random assumption, which states that the probability of a value being missing does not depend on the unobserved data conditional on the observed data. Although this assumption cannot be verified empirically, its plausibility can be increased by including many predictors of missingness [1].

**Imputation model**

The imputation methods used were predictive mean matching for continuous variables, binomial logistic regression for binary variables, and multinomial logistic regression for categorical variables with more than two levels. In predictive mean matching for some target variable, a missing value of a recipient is substituted by an observed value of a donor, where the donor is randomly drawn from patients whose expected values for the target variable conditional on the observed variables are within some predefined distance from that of the recipient [1].

All variables that appear in the complete case analysis, i.e., the confounders of the obesity-mortality relationship, as well as obesity and the mortality endpoint itself, should be included as predictors in the imputation model. Additional predictors to be included are variables that are related to the missingness and variables that explain a substantial proportion of variance in the target variable, provided that these variables contain few missing values within the subgroup of patients with missing data on the target variable [1].

Missing values were imputed for obesity, ethnicity, income, smoking status, alcohol consumption, physical activity, hypothyroidism, chronic glucocorticoid therapy, solid malignancy, hematological malignancy, dementia, human immunodeficiency virus infection or acquired immunodeficiency syndrome, and calendar time. The confounders of the obesity-mortality relationship were included as predictors in the imputation model, along with obesity and the mortality endpoint. Screening for predictors that had an absolute correlation with the target variable or its missingness indicator of at least 0.20 and that had at least 25% of usable cases (defined as the proportion of cases with missing data on the target variable that had observed values on the predictor) could identify following additional predictors: chronic respiratory disease for smoking status, chronic liver disease for alcohol consumption, Charlson comorbidity index for solid malignancy, and Charlson comorbidity index for physical activity. The number of iterations was set to 20 and the number of imputations to 50. Convergence of the MICE algorithm was assessed using trace plots and found appropriate.

**References**

1. van Buuren S, Groothuis-Oudshoorn K. mice: Multivariate Imputation by Chained Equations in R. Journal of Statistical Software. 2011;45(3):67.
