# Supplementary material for: The obesity paradox in critically ill patients: a causal learning approach to a casual finding
Source: Crit Care. 2020 Aug 5;24:485. doi: 10.1186/s13054-020-03199-5 (PMC7405433; doi:10.1186/s13054-020-03199-5)
Supplement: Supplementary file 3 — Additional file 3. Sensitivity analysis using different BMI cut-off values. [file 13054_2020_3199_MOESM3_ESM.docx]

**Additional File 3**

| **Table C1.** Summary table of the traditional versus robust causal inference approach for BMI cut-off of ≥25 kg/m^2^. | | | | | | | |
| --- | --- | --- | --- | --- | --- | --- | --- |
|  |  | **Complete cases in A** | | **Complete cases in (A, C)** | | **Imputation for (A, C)** | |
| **Estimand** | **Method** | **Estimate**  **(95% CI)** | **P** | **Estimate**  **(95% CI)** | **P** | **Estimate**  **(95% CI)** | **P** |
| **Raw marginal risk**  **difference (unadjusted)** | Regression | -1.40%  (-3.14% to 0.35%) | 0.117 | -2.45%  (-4.43% to -0.46%) | 0.016 | -1.36%  (-3.12% to 0.40%) | 0.130 |
| **Marginal causal risk**  **difference (ATU)** | Regression +  G-computation | **-** | **-** | -2.11%  (-4.10% to -0.12%) | 0.038 | -1.19%  (-2.95% to 0.57%) | 0.185 |
|  | TMLE +  super learner | **-** | **-** | -1.08%  (-3.06% to 0.90%) | 0.283 | -0.01%  (-1.81% to 1.79%) | 0.992 |
| with A=1 if BMI≥25 kg/m^2^, A=0 if BMI<25 kg/m^2^, and C=(age, sex, ethnicity, income, smoking status, alcohol consumption, physical activity, hypothyroidism, chronic glucocorticoid therapy, solid malignancy, hematological malignancy, dementia, human immunodeficiency virus/acquired immunodeficiency syndrome, calendar time).  Abbreviations: ATU, average treatment effect in the untreated; CI, confidence interval; TMLE, targeted maximum likelihood estimation. | | | | | | | |

| **Table C2.** Summary table of the traditional versus robust causal inference approach for BMI cut-off of ≥35 kg/m^2^. | | | | | | | |
| --- | --- | --- | --- | --- | --- | --- | --- |
|  |  | **Complete cases in A** | | **Complete cases in (A, C)** | | **Imputation for (A, C)** | |
| **Estimand** | **Method** | **Estimate**  **(95% CI)** | **P** | **Estimate**  **(95% CI)** | **P** | **Estimate**  **(95% CI)** | **P** |
| **Raw marginal risk**  **difference (unadjusted)** | Regression | 1.89%  (-2.19% to 5.97%) | 0.364 | 0.62%  (-3.90% to 5.13%) | 0.789 | 1.85%  (-2.19% to 5.90%) | 0.370 |
| **Marginal causal risk**  **difference (ATU)** | Regression +  G-computation | **-** | **-** | -0.65%  (-4.80% to 3.51%) | 0.760 | -0.12%  (-3.78% to 3.54%) | 0.949 |
|  | TMLE +  super learner | **-** | **-** | 0.46%  (-3.10% to 4.02%) | 0.802 | 0.90%  (-2.74% to 4.53%) | 0.629 |
| with A=1 if BMI≥35 kg/m^2^, A=0 if BMI<35 kg/m^2^, and C=(age, sex, ethnicity, income, smoking status, alcohol consumption, physical activity, hypothyroidism, chronic glucocorticoid therapy, solid malignancy, hematological malignancy, dementia, human immunodeficiency virus/acquired immunodeficiency syndrome, calendar time).  Abbreviations: ATU, average treatment effect in the untreated; CI, confidence interval; TMLE, targeted maximum likelihood estimation. | | | | | | | |
